# Targeting the Wnt/β-catenin pathway in human osteosarcoma cells

**DOI:** 10.18632/oncotarget.26377

**Published:** 2018-12-04

**Authors:** Fang Fang, Ashley VanCleave, Ralph Helmuth, Haydee Torres, Kirby Rickel, Hannah Wollenzien, Hongli Sun, Erliang Zeng, Jing Zhao, Jianning Tao

**Affiliations:** ^1^ Cancer Biology and Immunotherapies Group, Sanford Research, Sioux Falls, SD, USA; ^2^ BRIN Scholar from Dakota Wesleyan University, Sanford Research, Sioux Falls, SD, USA; ^3^ Department of Chemistry and Biochemistry, South Dakota State University, Brookings, SD, USA; ^4^ Department of Biomedical Engineering, University of South Dakota, Sioux Falls, SD, USA; ^5^ Basic Biomedical Sciences, University of South Dakota, Vermillion, SD, USA; ^6^ Department of Oral and Maxillofacial Surgery, University of Iowa, Iowa City, IA, USA; ^7^ Departments of Preventive & Community Dentistry, Biomedical Engineering, and Biostatistics, Division of Biostatistics and Computational Biology of College of Dentistry, University of Iowa, Iowa City, IA, USA; ^8^ Population Health Group, Sanford Research, Sioux Falls, SD, USA; ^9^ Department of Internal Medicine, University of South Dakota, Sioux Falls, SD, USA; ^10^ Department of Pediatrics, University of South Dakota, Sioux Falls, SD, USA

**Keywords:** osteosarcoma, Wnt/β-catenin signaling, PRI-724, Cyclin D1

## Abstract

Aberrant activation of Wnt signaling has been implicated in human osteosarcoma, which may provide a genetic vulnerability that can be targeted in osteosarcoma treatment. To test whether Wnt activation is necessary for osteosarcoma growth, colony formation, invasion, and metastasis, we treated human osteosarcoma cells with a small molecule inhibitor of Wnt/β-catenin, PRI-724, which suppresses Wnt/β-catenin-mediated transcription. We found increased protein levels of endogenous active-β-catenin in five human osteosarcoma cell lines. Treatment with PRI-724 was sufficient to inhibit human osteosarcoma 143B and SJSA-1 cell proliferation. Suppressed Wnt signaling was confirmed by decreased protein levels of the Wnt target Cyclin D1. Furthermore, we revealed significant inhibitory effects on cell migration, invasion, and colony formation in the human osteosarcoma cells. Using deposited data from next generation sequencing studies, we analyzed somatic mutations and gene expression of components in the Wnt/β-catenin pathway. We found somatic mutations and upregulated gene expression of many components in the Wnt/ β-catenin pathway, indicating activated Wnt signaling. Taken together, our results illustrate the critical role of Wnt/β-catenin signaling in human osteosarcoma pathogenesis and growth, as well as the therapeutic potential of Wnt inhibitors in the treatment of human osteosarcoma.

## INTRODUCTION

Osteosarcoma (OS) is the most common type of bone cancer in adolescents and young adults with an incidence rate of 4.4 per million people per year in the age group of 0-24 years for all races and both genders [1, 2]. Molecular genetic studies of osteosarcoma have improved our view of the etiology of the disease and therapeutic approaches for patients [2–4]. Current disease management strategies include surgical resection of all clinically visible tumors and systemic chemotherapy [5]. The five-year survival rate for localized osteosarcoma is about 70% while the survival rate for patients with metastatic or recurrent disease is less than 30% [6]. Moreover, the outcome for either group of patients with OS has not changed in several decades [7]. This highlights the need for new second-line treatment options for patients.

Recent studies have shown that osteosarcoma cancer stem cells play major roles in chemo-resistance, tumor recurrence, and metastasis [8, 9]. Cancer stem cells share overlapping features with normal stem cells and hijack developmental signaling pathways such as Wnt and Notch [10]. The Wnt signaling pathway is an evolutionarily conserved pathway that controls stem cell replication, survival, differentiation, calcium homeostasis, cell polarity, and adult tissue homeostasis including in the skeletal system [11–16]. The canonical Wnt/β-catenin signaling pathway, one of three major Wnt pathways, is the best understood. In the presence of Wnt ligands that bind to receptors, the accumulation of cytoplasmic β-catenin protein enables its translocation to the nucleus, where it induces cellular responses via transactivation of target genes such as *CCND1* [10]. Activation of canonical Wnt/β-catenin signaling serves as a genetic driver in many types of cancer, including colorectal, lung, breast, ovarian, prostate, liver, brain, synovial sarcoma, and Schwann cell tumor [12, 17, 18]. Studies that target Wnt/β-catenin signaling in Wnt-activation-associated cancers have opened new avenues for the development of effective agents that inhibit Wnt activation [10].

Deregulation of canonical Wnt/β-catenin signaling in human osteosarcoma samples and cell lines has been described in recent studies [19–23]; however, the role of activated Wnt/β-catenin signaling in the pathogenesis of osteosarcoma remains poorly understood. The development of therapeutic agents specifically targeting the aberrant Wnt activation in OS cells is still in its infancy. Recently, we presented evidence that Wnt/β-catenin signaling plays a potential role in osteosarcoma development in murine models of the disease [24]. To prove that Wnt activation is necessary for osteosarcoma growth, colony formation, invasion, and metastasis, we first treated human OS cells with PRI-724 (an ICG-001 derivative), a small molecule inhibitor of CBP (CREB binding protein)/β-catenin complex formation, which suppresses Wnt/β-catenin-mediated transcription [25]. Additionally, we investigated the extent of activated Wnt/β-catenin signaling in human osteosarcoma samples, whose genome or transcriptome were analyzed via high-throughput sequencing and bioinformatics approaches. We further provide evidence that constitutive Wnt/β-catenin signal activation is common in human osteosarcoma, while activated genetic mutations of the Wnt pathway components are rare. Altogether, our results form the first proof-of-concept study using the small molecule PRI-724 for inhibiting CBP-β-catenin binding to decrease human osteosarcoma cell growth. Our data illustrate the critical role of Wnt/β-catenin signaling in human osteosarcoma pathogenesis and metastasis and suggest Wnt/β-catenin components as promising therapeutic targets for the treatment of human osteosarcoma.

## RESULTS

### Human OS cells sustain high Wnt/ β-catenin signaling level

**Figure 1:**
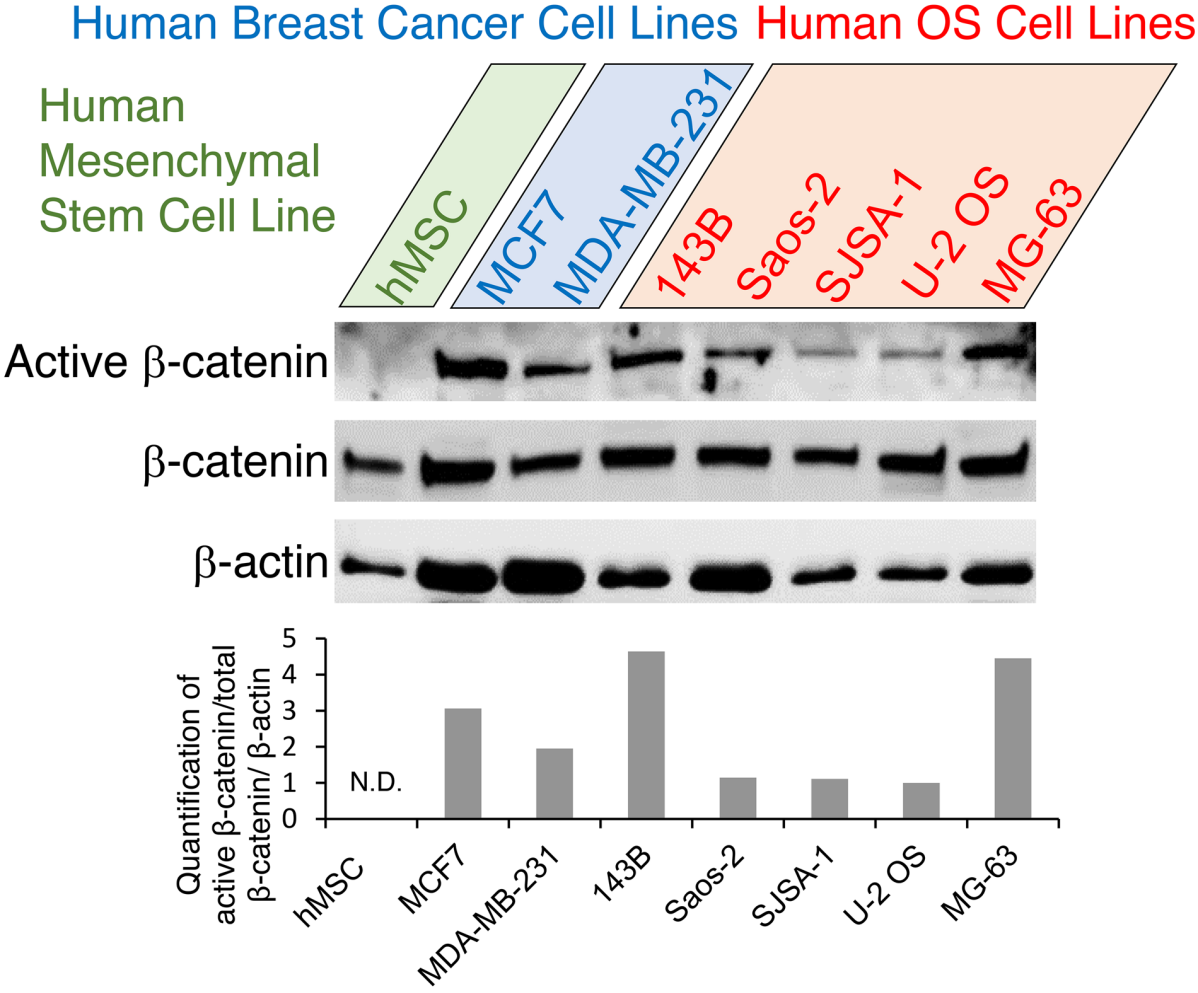
Expression analysis of Wnt pathway genes in human OS cells Western blot analysis of active β-catenin, total β-catenin and β-actin proteins in human OS cell lines (143B, Saos-2, SJSA-1 , U-2 OS, MG-63) with human breast cancer cell high-level-expression controls (MCF7, MDA-MB-231) and additional human mesenchymal stem cell low-level-expression controls (hMSC). Representative blots are shown in the top panels, and densitometric quantification of the blots is shown in the bottom graph. N.D., not detectable.

To gain a better understanding of Wnt activation signaling pathway in human OS cells, we first performed Western blotting to detect active β-catenin, total β-catenin, and β-actin protein levels in human OS cell lines 143B, Saos-2, SJSA-1 , U-2 OS, and MG-63. Breast cancer lines MCF7 and MDA-MB-231 were used as controls for high-level expression of active β-catenin protein whereas hMSC cell line was used as a control for low-level expression of active β-catenin protein. As shown in Figure 1, sustained, high Wnt/β-catenin signaling activity in human OS cells was represented by increased level of active β-catenin protein, with 143B showing the highest level. This prompted us to examine the potential therapeutic effects of novel Wnt inhibitors on human OS cells.

### PRI-724 inhibited human OS cell 143B proliferation

**Figure 2:**
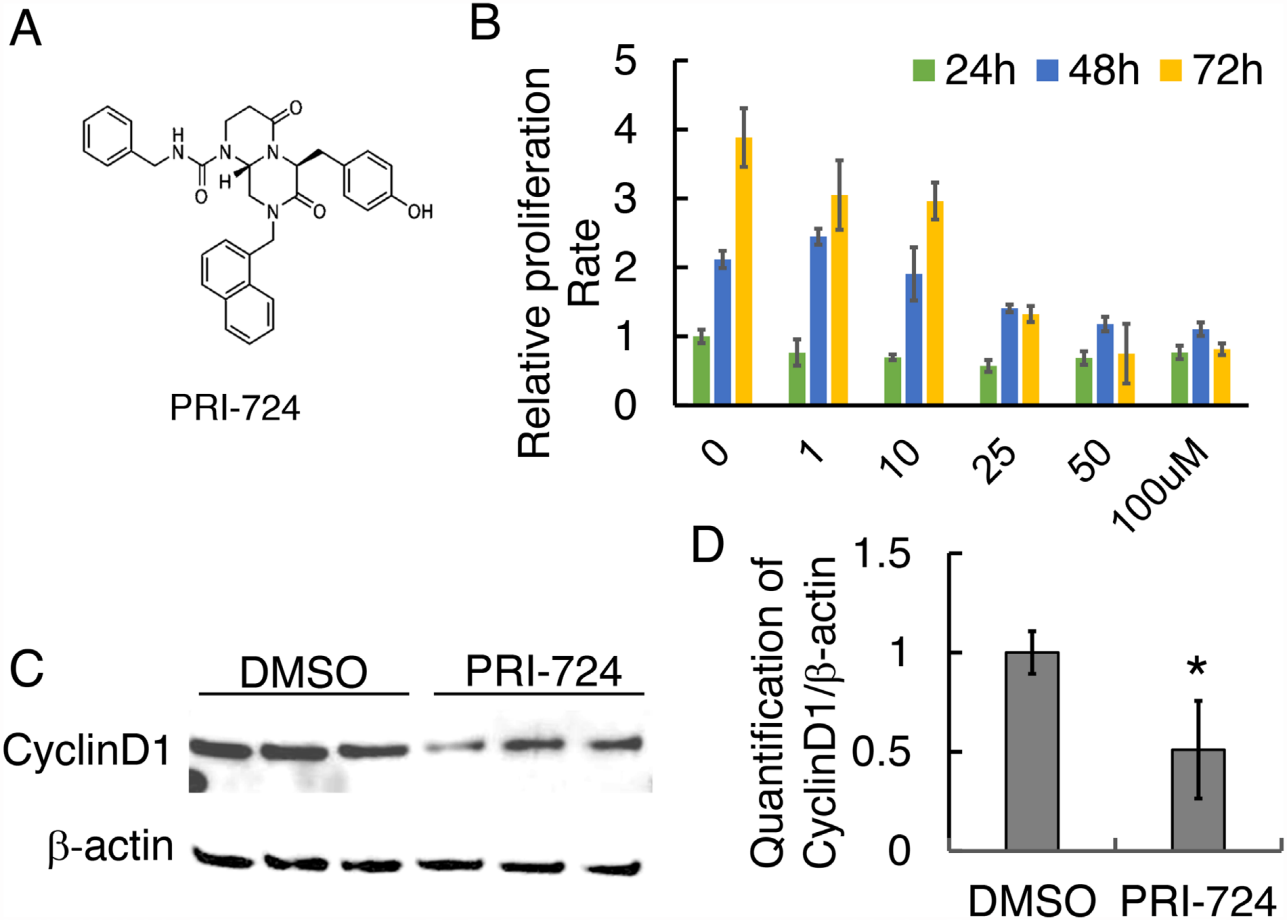
Effect of PRI-724 on 143B cell proliferation **(A)** Chemical structure of PRI-724 (modified from https://www.selleckchem.com/). **(B)** 143B cell proliferation assay upon PRI-724 treatment. **(C)** Western blotting shows CyclinD1 protein level following PRI-724 treatment in 143B cells. **(D)** Quantification of (C).

To test if Wnt activation is necessary for osteosarcoma cell proliferation, migration, invasion, and colony formation, we treated human osteosarcoma 143B cells with a small molecule inhibitor of the Wnt/β-catenin pathway, PRI-724 (also named ICG-001, Figure 2A). Effects of PRI-724 on many cancer cell lines have been studied and it has been applied in preclinical and clinical trials [25, 31–34]. However, the therapeutic effect of PRI-724 on OS is unknown. First, the proper working concentration of PRI-724 on 143B cells viability was examined using a cell proliferation assay. Among five different concentrations, 25 μM or higher of PRI-724 was enough to suppress 143B cell proliferation at all three time points of 24 hours (24h), 48h, and 72h (Figure 2B). To investigate the effect of PRI-724 on Wnt/β-catenin signaling activity, protein levels of Wnt canonical targets including Cyclin D1 and Survivin in cells treated with PRI-724 for 24 hours were tested using Western blot. As expected, Wnt signaling activity was significantly down-regulated in PRI-724 treated cells (Figure 2C, 2D; Supplementary Figure 1A & 1B).

### PRI-724 inhibited human OS 143B cell migration and invasion

**Figure 3:**
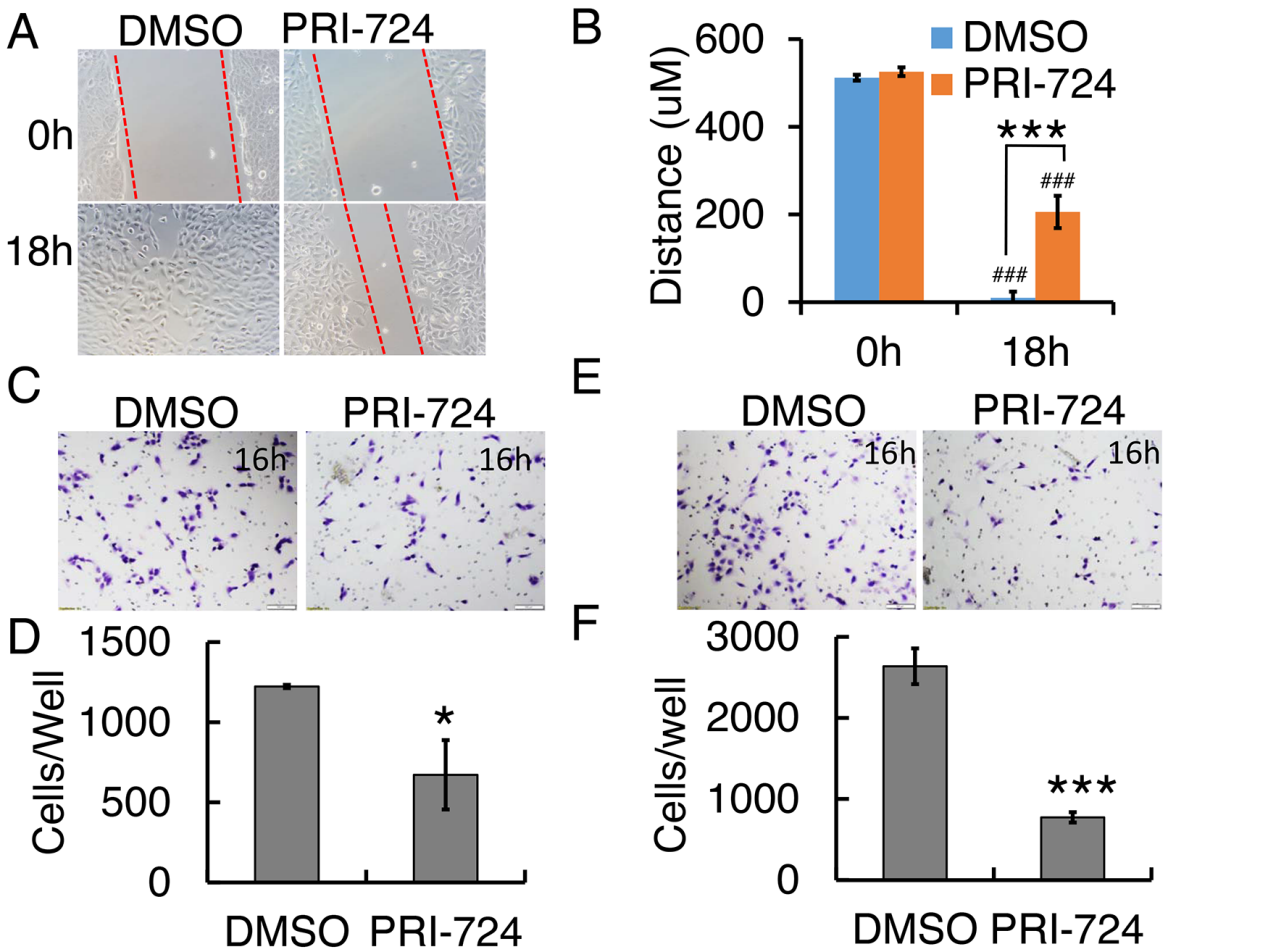
Effect of PRI-724 on 143B cell migration and invasion **(A)** 143B cell wound closure assay upon PRI-724 treatment. Red dashed lines indicate cell migration fronts. **(C)** 143B cell migration in Transwell system with or without PRI-724 treatment. **(E)** 143B cell invasion through Transwells coated with Matrigel, with or without PRI-724 treatment. **(B, D, F)** Quantification of (A, C, E).

In order to understand the effects of PRI-724 on cell migration, we first performed a wound healing assay. After 18h, PRI-724-treated 143B cells migrated significantly slower than those treated with DMSO vehicle solution (Figure 3A & 3B). To further examine this phenomenon, we employed a Transwell system to examine cell migration behavior under PRI-724 treatment. Quantification of cells that migrated through the insert membrane showed about 50% reduction under PRI-724 treatment (Figure 3C & 3D). To investigate cell invasion *in vitro*, the inserts in the Transwell system were coated with a layer of Matrigel to mimic the extracellular matrix of the *in vivo* tumor environment. Notably, PRI-724 greatly suppressed 143B cell invasion to about 30% of control (Figure 3E & 3F). Altogether, these data imply that inhibition of Wnt/ β-catenin canonical signaling with PRI-724 may suppress human tumor metastasis in human OS patients.

### PRI-724 inhibited human OS SJSA-1 cell proliferation and metastasis

**Figure 4:**
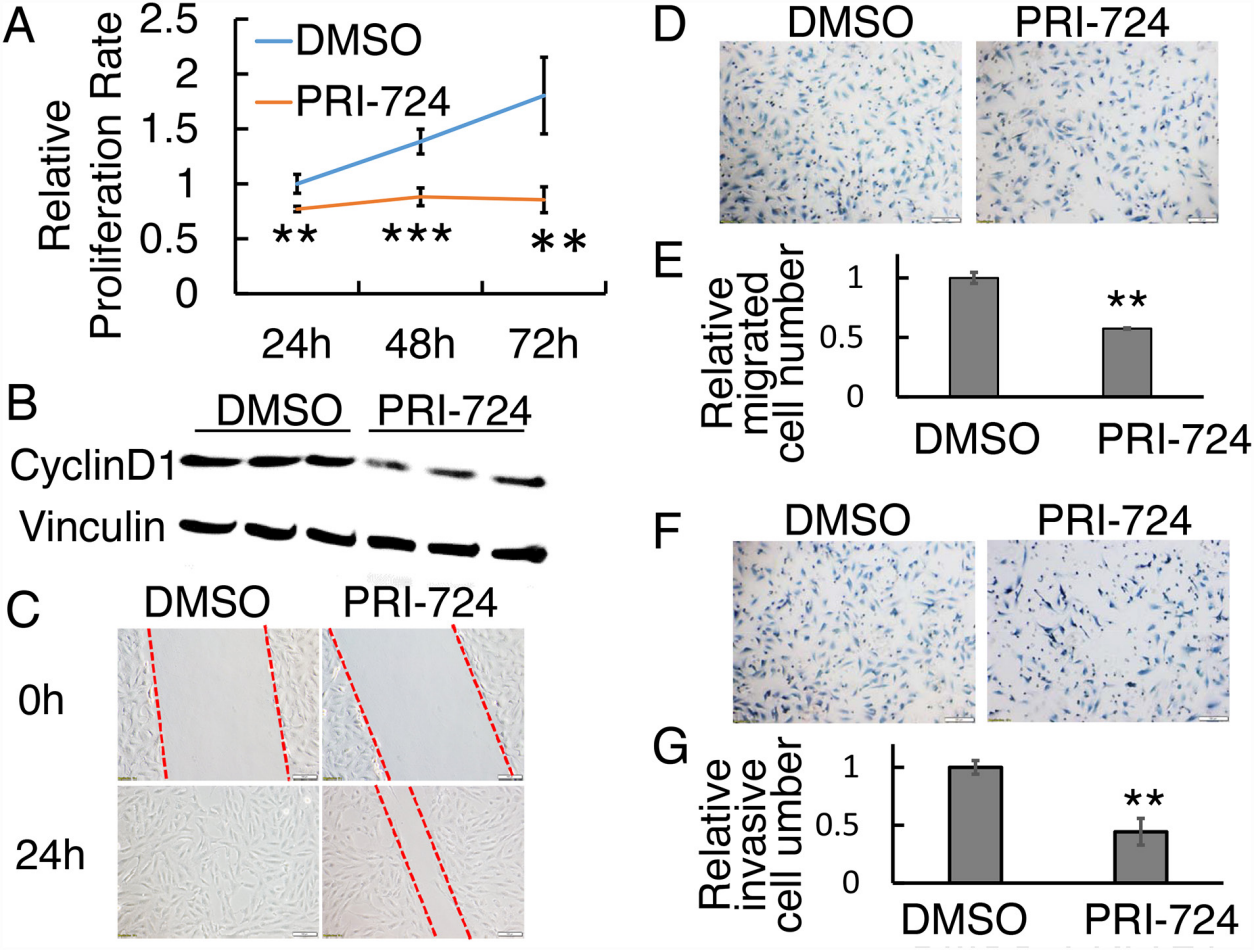
Effect of PRI-724 on SJSA-1 cell proliferation, migration, and invasion **(A)** SJSA-1 cell proliferation assay upon PRI-724 treatment. **(B)** Western blotting shows CyclinD1 protein level following PRI-724 treatment in SJSA-1 cells. **(C)** SJSA-1 cell wound closure assay upon PRI-724 treatment. Red dashed lines indicate cell migration fronts. **(D)** SJSA-1 cell migration in Transwell system with or without PRI-724 treatment. **(E)** Quantification of (D). **(F)** SJSA-1 cell invasion through Transwells coated with Matrigel, with or without PRI-724 treatment. **(G)** Quantification of (F).

To validate the observed drug effects on a second human OS cell line,, we treated SJSA-1 with PRI-724. An inhibitory effect of PRI-724 on SJSA-1 cell proliferation with a concentration of 25 μM was observed at 24h, 48h, and 72h (Figure 4A). Cyclin D1 and survivin protein levels in SJSA-1 cells were significantly reduced after 24h treatment (Figure 4B; Supplementary Figure 1C & 1D). PRI-724 suppressed SJSA-1 cell wound closure at 24 hours compared with the DMSO control treatment, in which the gap disappeared (Figure 4C). To dissect out the relative contribution from cell proliferation versus migration, SJSA-1 cell were starved for 16 hours before seeding into Transwell system with or without Matrigel to assess the cells’ ability to migrate and invade. Similar to 143B cells, 25 μM PRI-724 significantly suppressed SJSA-1 cell migration by about 40% (Figure 4D & 4E) and invasion by about 30% (Figure 4F & 4G), compared to control levels.

### PRI-724 inhibited colony formation of human OS cells

**Figure 5:**
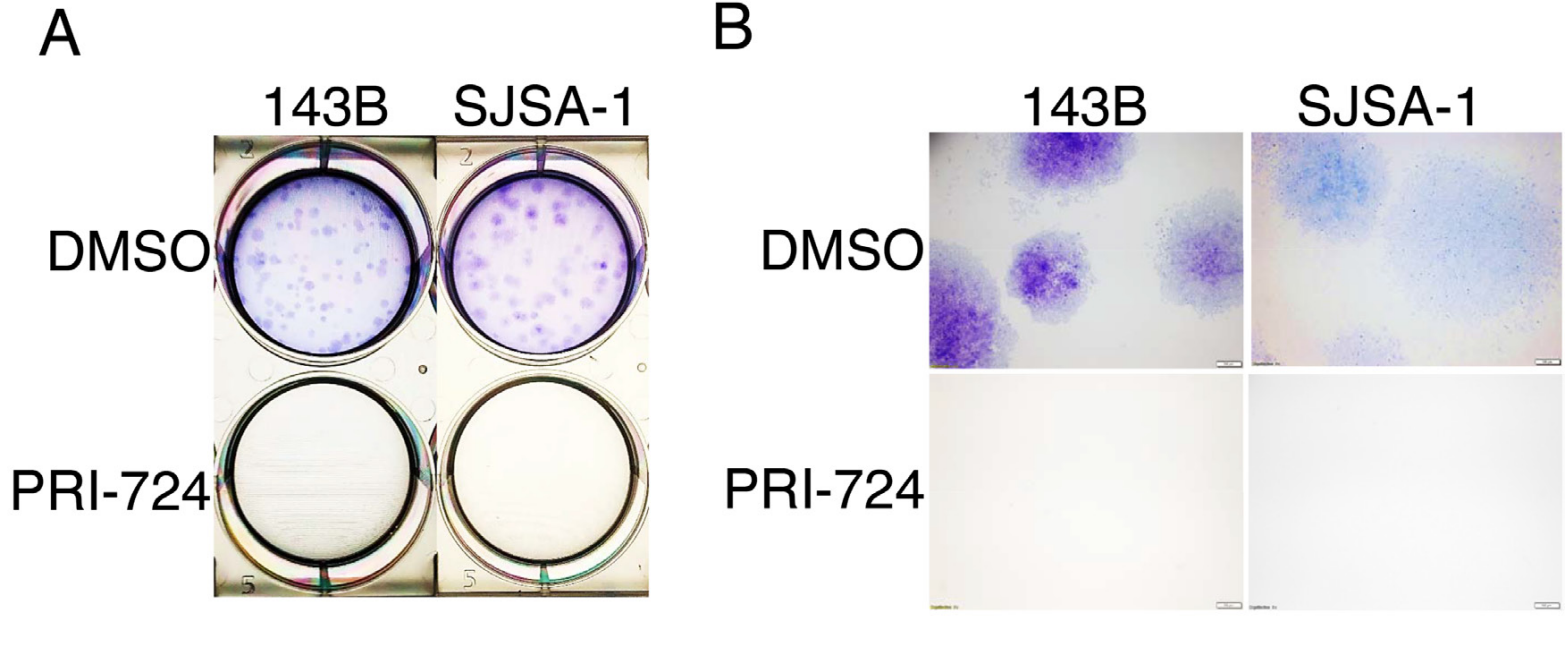
Effects of PRI-724 on 143B and SJSA-1 cell colony formation **(A)** Images of crystal violet stained 143B and SJSA-1 colonies in wells. **(B)** Images of colonies under 2X magnification.

Colony formation assay is an *in vitro* technique for detecting the ability of a single cell to grow into a large colony by clonal expansion. This assay can also be employed to evaluate the anti-proliferative activity of potential anti-tumor agents. Moreover, colony formation closely simulates the pathological process of tumor development *in vivo* [35]. To study the role of Wnt canonical signaling in osteosarcoma development, we performed a colony formation assay using 143B and SJSA-1 cells treated with PRI-724. Crystal violet staining clearly showed that treatment with 25 μM PRI-724 completely inhibited clonogenic ability of 143B and SJSA-1 cells (Figure 5A & 5B). Since clonogenic activity is a sensitive indicator of undifferentiated cancer stem cells (CSCs), this result implies that Wnt signaling may be required for CSC self-renewal and proliferation in order to maintain CSC population in tumor tissues.

### Deregulated expression and activating somatic mutations of Wnt/β-catenin pathway components in pediatric OS

**Figure 6:**
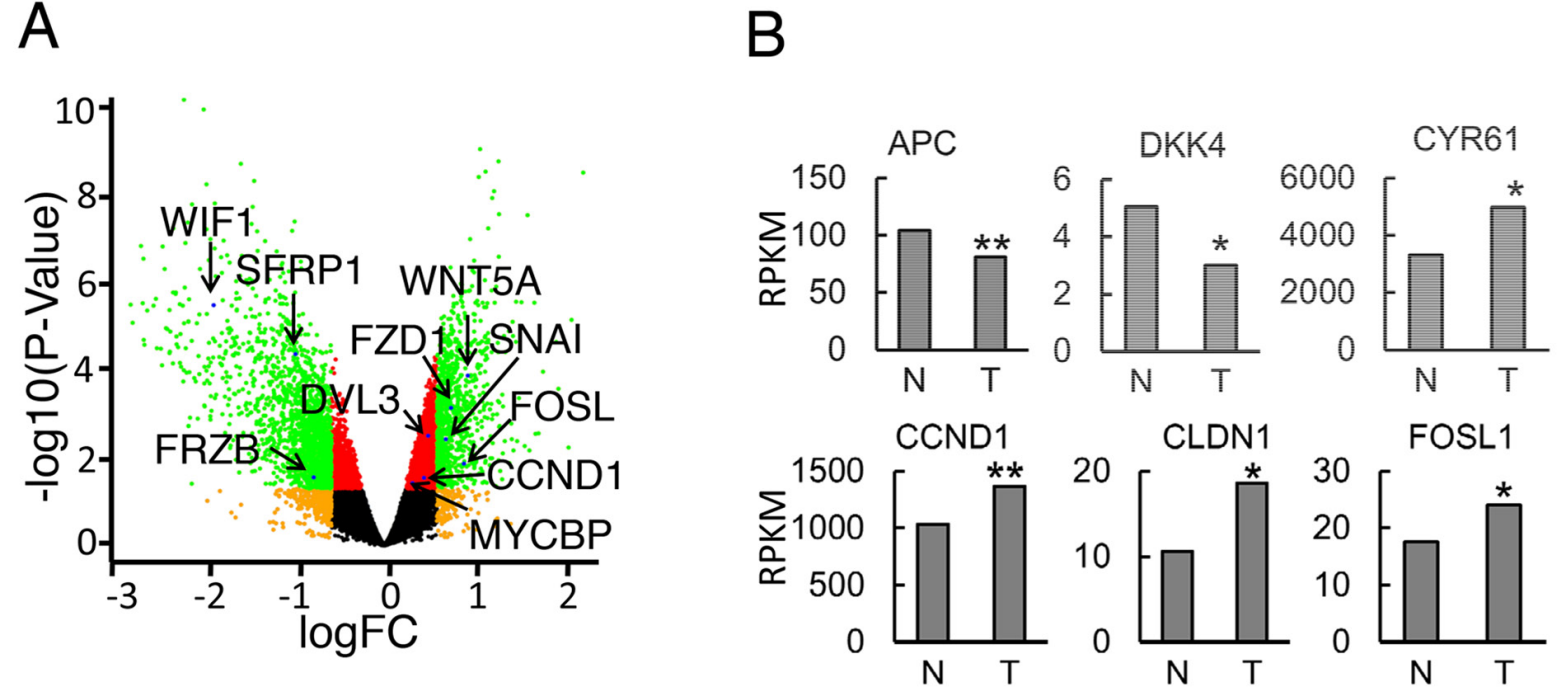
Gene expression of Wnt pathway components in human OS tumor samples **(A)** Volcano-plot analysis of gene expression in human OS and normal tissue control. (n=18 paired samples). Differential transcripts with statistical significance (FDR < 0.05, -log10 of p-value, y-axis) are shown in red (< 1.5-fold change) or green (> 1.5-fold change) dots. Non-significant genes (FDR > 0.05) are shown in black or orange dots (1.5-fold cutoff). Arrows label select genes with significant differential expression. **(B)** Median RPKM values of differentially expressed genes between OS and normal tissue control (n=18 paired samples, median, Wilcoxon signed-rank test, ^*^, P< 0.05; ^**^, P<0.01; ^***^, P<0.001).

To gain a better understanding of changes in individual gene expression of the Wnt signaling pathway in context of the whole transcriptome of human OS tumor tissues, we re-analyzed a recently published RNA-seq study of 18 patients (Dataset: GSE99671) [29]. We used paired samples that allowed a direct comparison of the diseased and normal tissue from the same patient. This design substantially reduces biological variability and increases statistical power [30]. Our bioinformatics analysis of paired human OS tumor and normal bone tissue samples using edgeR demonstrated significantly-altered (p < 0.05) expression of many Wnt-pathway-related genes (Supplementary Table 1). Many of them are Wnt direct target genes, overlapping with target genes listed on the Wnt homepage (https://web.stanford.edu/group/nusselab/cgi-bin/wnt/target_genes, last updated May, 2018) Among these altered genes, Wnt antagonists *FRZB, SFRP1*, and *WIF1* were decreased in tumor samples while Wnt ligands *WNT5A, FZD1, DVL3* and target transcription factors *CCND1, MYCB, SNAI2* were increased in tumor samples (Figure 6A). Moreover, we performed RPKM comparison through Wilcoxon signed-rank test and found decreased expression of Wnt inhibitor genes *DKK1* and *DKK4* and increased Wnt canonical target genes *CCND1, CLND1, and FOSL1* (Figure 6B). Among the upregulated Wnt target genes, *FOSL1* is an oncogene that has recently been discovered in lung cancer and pancreatic cancer [36]. In addition, survivin is an anti-apoptotic protein that regulates cell division [37]. We observed significantly increased expression of *BIRC5*, which encodes survivin protein (Supplementary Table 1). Consistently, PRI-724 induced significant downregulation of survivin in both 143B and SJSA-1 OS cells (Supplementary Figure 1A-1D), implicating that survivin may be responsible for inhibition of colony expansion in the colony formation assay (Figure 5). Together, our data suggest that hyper-activated Wnt/ β-catenin signaling may be a common mechanism of OS genesis in patients.

Next generation sequencing (NGS), using either whole-genome sequencing or exome sequencing of human OS samples, has helped us decipher the genetic causes of OS at a much higher resolution and in an unbiased fashion. In the past few years, several groups have performed NGS analysis on a total of 111 human OS samples and their normal tissue pairs [3, 38–43]. This analysis has expanded the somatic mutation catalogue of human OS. Among a few hundred of the mutations found in those samples, many components of the Wnt signaling pathway have been identified in one or more samples. We re-analyzed all somatic mutations related to Wnt pathway genes from three studies (Chen et al., 20 WGS [38]; Perry et al., 59 WES [44]; and Kovac et al., 31 WES [41]). Somatic mutation types and frequency that occurred in more than one study are summarized in Table 1. Those genes include *MYC, APC, CTNND1, CCNE1, CDK4, NF1, DLG2,* and *BAP1.* Mutation types include copy number alteration (amplified or deleted), in frame deletion, structural variation, frame shift, copy gain/loss, and point mutations (e.g. missense mutation). For example, copy number amplification of the oncogene *MYC* has been found in most types of cancer [45, 46]; copy number loss of the tumor suppressor *APC* may lead to increased nuclear functional β-catenin protein in OS [47–49]. The human data is supported by a recent study from Natacha et al. where a spontaneous mouse model of OS with *Apc*1638N/+;*twist* null/+ mutation mimics the human disease [50]. However, mutation frequencies of Wnt related genes are rather low or rare. Taken together, this NGS data sheds light on the importance of Wnt signaling in OS initiation, progression, and metastasis, implying Wnt canonical pathway as a potential drug target for OS treatment.

**Table 1:** Somatic mutations of Wnt pathway components in human OS

Genes	Mutation types and frequency (Reference)
CDK4	Copy Number Amplified 1/20 (Perry et al. [44]) (Kovac et al. [41])
MYC	Copy Number Amplified 2/20 , In Frame Deletion p.Q37del 1/20 (Perry et al. [44])
GNAS	Structural Variation 2/34 (Chen et al. [38])
APC	Copy Number Loss (Kovac et al. [41])
CTNND1	Copy Number Gain (Kovac et al. [41])
CCNE1	Copy Number Amplified 2/20 (Perry et al. [44]) Copy Number Gain (Kovac et al. [41])
NF1	Structural Variation 2/34 (Chen et al. [38]), Copy Number Deletion 2/20, p.S2309fs frame shift deletion (Perry et al. [44])
DLG2	Structural Variation 18/34 (Chen et al. [38]), Copy Number Deletion 2/20, p.E481K missense mutation 1/20, p.H27Y missense mutation, p.N96fs frame shift deletion (Perry et al. [44]), Copy Number Loss (Kovac et al. [41])
BAP1	Copy Number Loss 38% (Kovac et al. [41])

## DISCUSSION

**Figure 7:**
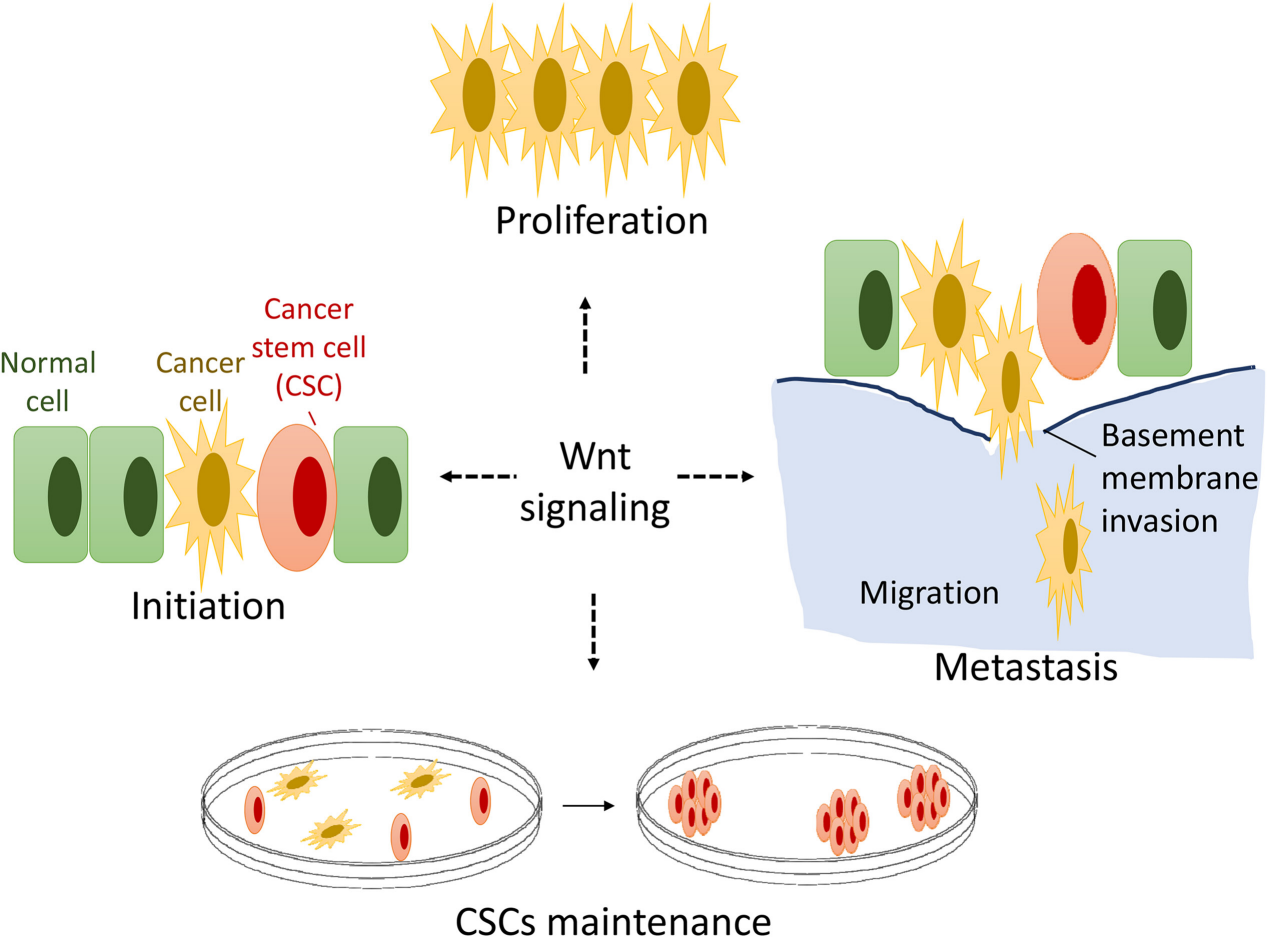
A hypothetical model of Wnt signaling in OS genesis and development Constitutive Wnt/β-catenin signal activation is common in human osteosarcoma while activating genetic mutations of Wnt pathway components in osteosarcoma are rare. Wnt/β-catenin signaling may play critical roles in OS proliferation, metastasis and OS cancer stem cell (CSC) maintenance.

Skeletal genetic studies in both humans and mice have established the Wnt/β-catenin signaling pathway as a required mechanism for osteoblast lineage determination, osteoblast differentiation, and proliferation [11, 13, 15]. However, how osteoblast lineage cells transform to osteosarcoma CSCs is poorly understood [9, 51]. Emerging evidence supports a crucial role for the Wnt/β-catenin signaling pathway in the development of OS, but there are two opposing hypotheses [3, 52, 53]. The first hypothesis is that inactivation of the Wnt/β-catenin pathway activity might contribute to osteosarcoma development [21, 22]. In contrast, other groups have suggested a second hypothesis, that activation of the Wnt/β-catenin signaling pathway drives osteosarcoma proliferation and growth and may serve as a potential novel therapeutic target [19, 20, 54]. We herein report the results of the first proof-of-concept study targeting Wnt/β-catenin by applying the small molecule compound PRI-724 to inhibit human osteosarcoma cell proliferation, migration, invasion, and clonogenic ability. Our results show that most OS cell lines and human OS tissues present hyperactive Wnt/β-catenin signaling activity relative to hMSCs, which exhibit low levels of endogenous Wnt signaling (Figure 1). Our data is consistent with the reports supporting the second hypothesis that activated Wnt/β-catenin pathway might contribute to osteosarcoma development and renewal of the CSCs [19, 20, 54, 55]. Our findings, along with those from those other groups, are consistent with the model that a hyperactive Wnt/β-catenin pathway may be required for OS proliferation, metastasis, and CSC maintenance and that activating mutations of the Wnt pathway may serve as initiating drivers in a small percentage of human OS [19, 20, 54, 55] (Figure 7). However, each aspect of this hypothetical model needs to be firmly defined in future studies, specifically using *in vivo* animal models to determine whether activated Wnt signaling has a causative role in OS development (Figure 7).

A growing body of evidence links Wnt/β-catenin signaling to tumorigenesis of many cancer types and implicates its role in the development of cancer drug resistance [56]. Due to the urgent requirement of inhibition of Wnt/β-catenin signaling in cancer therapy, a large amount of effort has been dedicated to developing therapeutic reagents that target this pathway, including both biological and small molecular agents. Currently, there are a number of clinical trials using potential therapeutic agents that target the Wnt/β-catenin pathway, including: OMP18R5, OMP-54F28, LGK974, CWP232291, Foxy-5, DKN-01, CGX1321, SM04755, ETC-1922159, OTSA101, OMP-54F28, SM04690, and PRI-724 [10, 57]. Among these agents, PRI-724 (a second-generation compound of ICG-001 and a low molecular weight inhibitor) binds specifically to CBP to disrupt the interaction between CBP and β-catenin, which leads to downregulation of β-catenin/TCF responsive gene expression, and induces apoptosis in colon cancer cells but not normal colonic epithelia cells [25]. In addition to colon carcinoma cells, PRI-724 has also been reported to exert anti-cancer effects on glioma cells [58, 59], gastric cancer cells [31] and leukemia [32]. PRI-724 has also been shown to improve chemo-resistance when combined with other drugs in hepatocellular carcinoma [33] and breast cancer [34]. Encouraging results from a study on safety and preliminary efficacy of anti-fibrotic activity using PRI-724 in patients with Hepatitis C Virus-related Cirrhosis in Phase 1 Trial has also been recently reported [60]. This study showed an undetectable influence of PRI-724 treatment on bone metabolism, which is interesting given that the Wnt signaling pathway has been shown to be crucial in bone development and homeostasis. Currently, PRI-724 has been applied in several clinical trials among many malignant cancers, including colon cancer, pancreatic cancer and leukemia (NCT01764477, NCT01302405, NCT01606579, and NCT02413853). In clinical trials, PRI-724 was applied in doses of up to 905 mg/m^2^/day (NCT01764477), corresponding to a concentration of 55 μM. Thus, the ICG-001 concentrations used in the present study (25 μM) *in vitro* may be achievable in patients. Our study using PRI-724 in human OS cell lines may pave the way to additional preclinical studies in mice and clinical trials on efficacy of the drug in patients with OS.

The molecular mechanisms of action of PRI-724, which serves as a cytostatic drug, have been explored over the past 14 years among many types of cancer cells [25, 31-34, 58, 59]. Distinct roles have been reported for CBP and for the related transcriptional coactivator p300, suggesting that CBP/β-catenin-mediated transcription is critical for proliferation/non-differentiation whereas p300/β-catenin-mediated transcription initiates differentiation [61]. Since PRI-724 binds specifically to CBP but not to p300 [25], PRI-724 likely has minimal impact on normal osteoblast and osteosarcoma cell differentiation. Indeed, the recent clinical trial showed undetectable effect of PRI-724 treatment on bone metabolism in patients [60]. Studies on the therapeutic efficacy of PRI-724/ICG-001 in pancreatic cancer have revealed one potential mechanism that Wnt/β-catenin-dependent inhibition of cell proliferation was partially due to downregulation of various genes that are involved in cell cycle, e.g. *CCNE1*, *E2, and A2* [59, 62]. In the present study, we found downregulation of Wnt canonical target Cyclin D1 and Survivin in both 143B and SJSA-1 cells after treatment with PRI-724 (Figure 2C, 4B; Supplementary Figure 1), suggesting that PRI-724 acts in a Wnt-dependent manner in the context of osteosarcoma. However, our study cannot exclude the possibility that PRI-724 may also inhibit OS cancer phenotypes in a Wnt-independent manner, which has been studied in cells such as pediatric high-grade gliomas and multiple myeloma [59, 63]. PRI-724 inhibition rate reaches a plateau at 25-100 μM (Figure 2B), suggesting additional mechanism(s) of action to be studied in the future. Moreover, PRI-724 combination therapy with other drugs such as chemotherapeutic agents warrants further study as Wnt plays a role in chemo-resistance [33, 34].

CSCs are responsible for cancer metastasis and reoccurrence, and cannot be easily eliminated by regular anti-proliferative drugs [64]. The clonogenic ability is one of many properties owned by CSCs, which we are just beginning to understand. The inhibitive effect of PRI-724 on human OS cells in this study warrants future studies on how the Wnt signaling pathway regulates stemness properties of OS CSCs. On the other hand, although a substantial body of evidence underscores the importance of the active Wnt/β-catenin pathway in the development of OS, so far there is only one available mouse model (*Apc;twist* deficit) based on mutations of Wnt pathway [50]. Moreover, how the APC mutation drives OS formation is still unknown. Therefore, future mouse genetic studies will not only illustrate the mechanisms of action of Wnt signaling in OS genesis or metastasis, but also provide animal models that can be used in preclinical studies of potential drugs including PRI-724 for the treatment of OS.

## MATERIALS AND METHODS

### Cell culture, inhibitor treatment, and Western blot analysis

Cell lines MCF7(HTB-22), MDA-MB-231(HTB-26), 143B(CRL-8303), Saos-2(HTB-85), SJSA-1 (CRL-2098), U-2 OS (HTB-96), and MG-63 (CRL-1427) were all purchased from ATCC and maintained in growth medium containing 10% Fetal Bovine Serum (FBS) (Fisher Scientific, ES009B) and 1% Penicillin-streptomycin (Hyclone, SV30010) at 37°C under a humidified atmosphere containing 5% CO_2_. Unless stated otherwise, cells in 100 mm tissue culture dishes (Fisher Scientific, 430167) were treated with 25 μM PRI-724 (Selleck, S8262) for 24 hours. Human mesenchymal stem cell (hMSCs) (Lonza, Walkersville, MD, USA) were cultured in Minimum Essential Medium (Gibco, Invitrogen, USA), containing 10% FBS (Gibco, Invitrogen, USA) and 100 U/ml penicillin, 100 mg/ml streptomycin sulfate (Gibco, Grand Island, NY, USA). The corresponding amount of DMSO (vehicle) was used as a control. Western blotting was performed according to the modified procedure described previously [26]. Briefly, cells were lysed by addition of 1X Laemmli Sample Buffer (Bio-rad, #1610737). Equal amounts of protein from each treatment group were separated o 4–20% Mini-PROTEAN® TGX™ Precast Protein Gels (Bio-rad, #4561094). Proteins were transferred onto PVDF membrane (Bio-Rad, # 1704272) using a semi-dry Bio-Rad Trans-blot apparatus. The membrane was then probed with one of the following primary antibodies: mouse anti-Active-β-Catenin (Anti-ABC) (Millipore, 05-665), rabbit anti-total β-Catenin (GeneTex, GTX101435), rabbit anti-Cyclin D1 (Cell Signaling, #2922), mouse anti-survivin, and anti-β-actin (Santa Cruz, sc-17779 and sc-47778, respectively), and mouse anti-Vinculin (Sigma-Aldrich V4505). The following secondary antibodies were used: horseradish peroxidase-conjugated Goat anti-rabbit (Fisher Scientific, PI31460), and Goat anti-mouse (Fisher Scientific, PI31436). Proteins were detected using a chemiluminescent substrate reagent kit (Fisher Scientific, 45-002-401) and were quantified with the optical density function of ImageJ software (NIH, Bethesda, MD, USA).

### Cell proliferation assay

Cells were seeded in a 96-well-plate (Fisher Scientific, N8010560) at a density of 2,000 cells per well and treated with PRI-724 at 0, 1, 10, 25, 50, or 100 μM for 24, 48 or 72 hours. 20 μl of 20 μM Sytox Green (Fisher Scientific, S7020) was added into each well. The plate was incubated in the incubator for 15 mins and read by plate reader SpectamaxM5 (Molecular devices, MV02017) at ex/em 485/530 with a 515 nm cut off for dead cell reading. 20 μl of 6% Triton X-100 (Boston BioProducts, P-925) was added to each well and the plate was incubated in the incubator for 45 mins and was read again for total cell reading. The value of total cell reading subtracted by the dead cell reading was considered as viable cell reading [27]. All experiments were performed with at least three independent replicates. P values < 0.05 were considered as significant and determined by Student’s t-tests.

### Wound healing assay

Cells were seeded in six-well plates and allowed to grow to confluence. Using a sterile 200-μl pipette tip, a straight scratch was drawn on the monolayer of cells. The cells were then washed three times with phosphate buffered saline (PBS). Fresh growth medium containing 25 μM PRI-724 or DMSO was added, and the cells were allowed to close the wound. Photographs were taken later at the same position of the wound. The width of the scratch wound was measured with NIH ImageJ. The relative migration at an indicated time point was calculated by comparing to the wound width at the initial time point.

### Cell migration and invasion assays

The *in vitro* migration and invasion assays were carried out using the 8-μm pore-sized Transwell chamber system (Fisher Scientific, 08-771-21). Prior to loading the cells into the upper chamber, the lower chamber was filled with medium containing 10% FBS with 25 μM PRI-724 or DMSO. Cells were starved overnight and added to serum-free medium containing 25 μM PRI-724 or DMSO in the upper chamber at a concentration of 4X10^4^ cells per well. For invasion assays, membranes were coated with a layer of 0.2 mg/ml Matrigel (Fisher Scientific, CB354248). After incubation for 24 hours, the cells on the upper surface of the well were removed completely by a Q-tip. The wells were fixed in 10% formalin and stained with Crystal violet (Fisher Scientific, C581-25). The plate with inserts were then imaged with an upright microscope (Olympus, IX71) and the relative number of cells that migrated through the Transwell pores was quantified using NIH ImageJ (NIH, Bethesda, MD, USA) [28].

### Colony formation assay

Cells were seeded in six-well plates at a density of 200 cells per well and cultured in full medium containing 25 μM PRI-724 or DMSO. Cells were grown for 7 to 14 days in a humidified, 5% CO_2_ atmosphere at 37°C until there was visible clonal colony formation. Images were taken after the colonies were washed gently with PBS (0.01 mol/L, pH 7.4), fixed with 10% formalin, and stained with 0.5% Crystal violet solution. Assays were performed at least three times.

### Gene expression analysis and statistical analysis

We used published data (GEO accession dataset: GSE99671) [29] from 18 human OS patients with paired normal bone tissue samples. edgeR package was used to perform differential gene expression analysis of the RNA-seq data with consideration of pair factor as described previously [30]. Comparison of Reads Per Kilobase Million (RPKM) between normal and tumor samples was performed using the Wilcoxon signed-rank test for paired data. Benjamini–Hochberg procedure was applied to adjust p values for multiple comparisons. A two-tailed Student’s t-test was used to compare two groups (PRI-724 vs. DMSO) in quantifications of Western blot, proliferation assays, wound healing assay, and Transwell migration and invasion assays. Results were considered significant at p < 0.05.

## SUPPLEMENTARY MATERIALS FIGURE AND TABLE


