# Supplementary material for: Targeting the Wnt/β-catenin pathway in human osteosarcoma cells
Source: Oncotarget. 2018 Dec 4;9(95):36780–92. doi: 10.18632/oncotarget.26377 (PMC6298399; doi:10.18632/oncotarget.26377)
Supplement: Supplementary file 1 [file oncotarget-09-36780-s001.pdf]

Targeting the Wnt/β-catenin pathway in human osteosarcoma cells

SUPPLEMENTARY MATERIALS

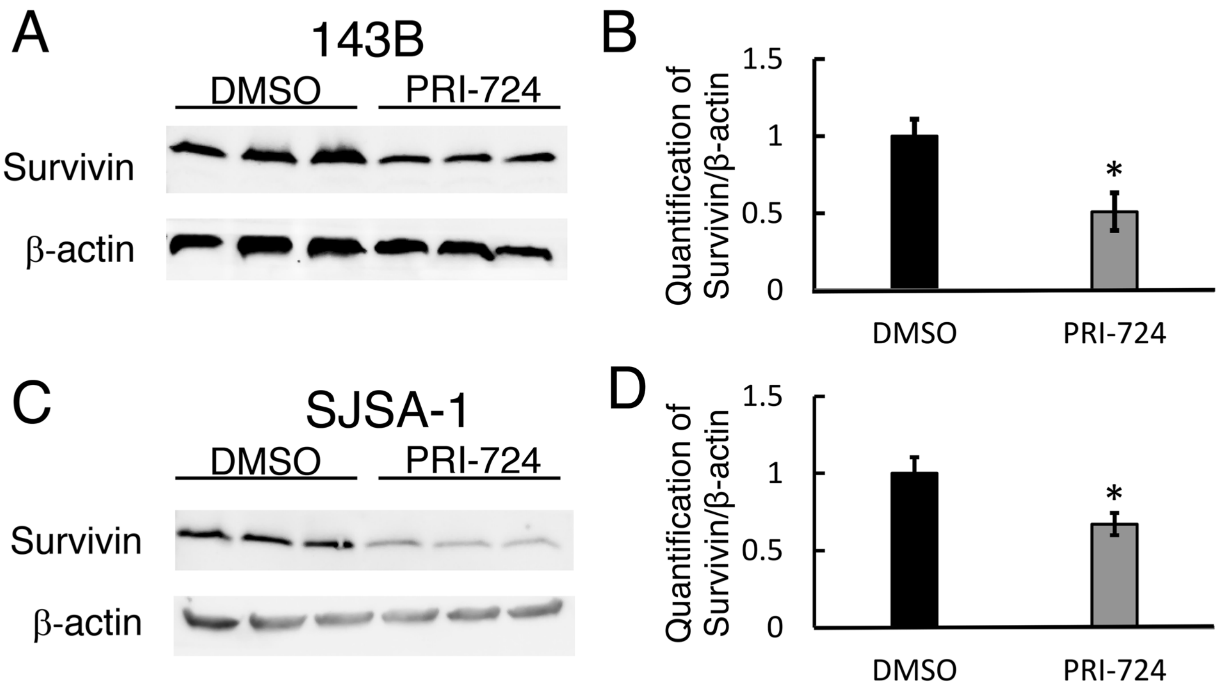

Supplementary Figure 1: Effects of PRI-724 inhibition on 143B and SJSA-1 cells. **(A)** Western blotting shows Survivin protein level of PRI-724 treatment in 143B cells; **(B)** Quantification of (A); **(C)** Western blotting shows Survivin protein level of PRI-724 treatment in SJSA-1 cells. **(D)** Quantification of (C).

Supplementary Table 1: Significantly (p<0.05) altered expression of Wnt-related genes

Symbol	logFC	logCPM	LR	PValue	FDR
WIF1	-1.878834	6.438416	21.676302	0.000003	0.000538
PTTG2	-1.344572	0.465160	9.149744	0.002488	0.029715
FGF9	-1.261956	3.221126	24.273959	0.000001	0.000272
CDH1	-1.221398	2.952594	9.488570	0.002068	0.026302
CLDN11	-1.010727	2.698215	17.767669	0.000025	0.001755
SFRP1	-0.978173	5.810134	16.751837	0.000043	0.002343
TGFBR3	-0.909750	5.546278	19.166080	0.000012	0.001209
WNT4	-0.876760	1.697744	13.275097	0.000269	0.007363
ABCB1	-0.863450	3.622305	11.946714	0.000547	0.011205
FRZB	-0.776031	6.683789	4.829488	0.027977	0.129941
ABCB10	-0.746416	5.170476	10.531181	0.001174	0.018222
FZD8	-0.737680	4.915936	6.154316	0.013109	0.081482
SNAI3	-0.656536	0.846251	6.883508	0.008699	0.063062
SOX15	-0.613973	0.487401	4.710907	0.029972	0.136135
TGFB2	-0.589627	6.384701	8.096411	0.004435	0.042150
SOX6	-0.550953	5.338293	4.323876	0.037581	0.155313
FZD5	-0.512032	6.022647	7.444599	0.006363	0.052742
FZD4	-0.505489	7.092100	4.936782	0.026291	0.125790
LRP5	-0.485558	3.550421	6.291177	0.012134	0.077449
NR5A2	-0.472007	4.591469	5.049658	0.024631	0.120942
TCF7L1	-0.420270	4.611241	4.013120	0.045148	0.174592
ACVR2B	-0.368300	5.406153	5.133184	0.023473	0.117802
TGFBR2	-0.330117	8.907449	4.083649	0.043300	0.170056
MYCBP	0.311751	5.600348	4.312455	0.037834	0.156040
GSS	0.404462	4.337865	6.918793	0.008529	0.062329
RARG	0.405429	3.644217	4.082091	0.043340	0.170155
EMP3	0.423969	4.367170	5.169430	0.022988	0.116394
FSTL1	0.425899	9.500477	4.490563	0.034082	0.146714
GJA1	0.435023	9.491574	3.883779	0.048755	0.183009
**CCND1**	**0.436204**	**8.627185**	**4.813138**	**0.028244**	**0.130698**
**AKT1**	**0.445993**	**5.017661**	**8.019398**	**0.004628**	**0.043188**
DVL3	0.482916	7.336485	8.743112	0.003108	0.033783
CYR61	0.514707	8.461440	4.253415	0.039171	0.159404
DKK3	0.517136	7.402351	4.366340	0.036656	0.153214
FZD2	0.535617	1.079652	6.151189	0.013132	0.081550
**SOX9**	**0.542783**	**5.915226**	**4.825323**	**0.028045**	**0.130100**
BIRC5	0.647137	4.611405	4.763730	0.029065	0.133218

(*Continued*)

Symbol	logFC	logCPM	LR	PValue	FDR
PLAUR	0.661539	5.189478	6.920143	0.008523	0.062304
SNAI2	0.678153	8.275258	8.394533	0.003764	0.038016
**FN1**	**0.710352**	**12.809494**	**9.456208**	**0.002104**	**0.026602**
CTLA4	0.715737	1.641120	4.662785	0.030823	0.138299
**EPHB3**	**0.724375**	**2.723639**	**8.679117**	**0.003219**	**0.034654**
**FZD1**	**0.735633**	**6.438397**	**11.453411**	**0.000714**	**0.013077**
**VEGFA**	**0.811289**	**6.310601**	**9.128724**	**0.002516**	**0.029921**
PITX2	0.829682	2.427778	7.584059	0.005889	0.050215
**TGFB3**	**0.832218**	**6.801064**	**7.545551**	**0.006016**	**0.050977**
FOSL1	0.878261	2.387146	6.107617	0.013460	0.082922
**WNT5A**	**0.919399**	**6.013088**	**14.611302**	**0.000132**	**0.004528**
**CDKN2A**	**1.014000**	**2.625011**	**6.424822**	**0.011254**	**0.074031**
EPHB2	1.135221	3.855004	13.770961	0.000207	0.006082

Notes: LogFC is the log fold-change; LogCPM are the log counts per million; LR is a likelihood ratio; Pvalue is a p-value; FDR is false discovery rate. Bolding indicates genes presented in Figure 6A.
